# Fetomaternal Expression of Glucose Transporters (GLUTs)—Biochemical, Cellular and Clinical Aspects

**DOI:** 10.3390/nu14102025

**Published:** 2022-05-12

**Authors:** Rafal Sibiak, Katarzyna Ozegowska, Ewa Wender-Ozegowska, Pawel Gutaj, Paul Mozdziak, Bartosz Kempisty

**Affiliations:** 1 Department of Histology and Embryology, Poznan University of Medical Sciences, 60-701 Poznan, Poland; rafal.sibiak@student.ump.edu.pl; 2 Doctoral School, Poznan University of Medical Sciences, 60-701 Poznan, Poland; 3 Department of Infertility and Reproductive Endocrinology, Poznan University of Medical Sciences, 61-701 Poznan, Poland; katarzyna.ozegowska@ump.edu.pl; 4 Department of Reproduction, Poznan University of Medical Sciences, 61-701 Poznan, Poland; ewozegow@ump.edu.pl (E.W.-O.); pawelgutaj@ump.edu.pl (P.G.); 5 Prestage Department of Poultry Science, North Carolina State University, Raleigh, NC 27695, USA; pemozdzi@ncsu.edu; 6 Department of Anatomy, Poznan University of Medical Sciences, 60-701 Poznan, Poland; 7 Department of Veterinary Surgery, Institute of Veterinary Medicine, Nicolaus Copernicus University in Torun, 87-100 Torun, Poland

**Keywords:** diabetes, glucose transporter proteins, hyperglycemia in pregnancy, placenta, pregnancy

## Abstract

Several types of specialized glucose transporters (GLUTs) provide constant glucose transport from the maternal circulation to the developing fetus through the placental barrier from the early stages of pregnancy. GLUT1 is a prominent protein isoform that regulates placental glucose transfer via glucose-facilitated diffusion. The GLUT1 membrane protein density and permeability of the syncytial basal membrane (BM) are the main factors limiting the rate of glucose diffusion in the fetomaternal compartment in physiological conditions. Besides GLUT1, the GLUT3 and GLUT4 isoforms are widely expressed across the human placenta. Numerous medical conditions and molecules, such as hormones, adipokines, and xenobiotics, alter the GLUT’s mRNA and protein expression. Diabetes upregulates the BM GLUT’s density and promotes fetomaternal glucose transport, leading to excessive fetal growth. However, most studies have found no between-group differences in GLUTs’ placental expression in macrosomic and normal control pregnancies. The fetomaternal GLUTs expression may also be influenced by several other conditions, such as chronic hypoxia, preeclampsia, and intrahepatic cholestasis of pregnancy.

## 1. Introduction

Pregnancy is a state of progressive adaptations across multiple maternal systems, including the cardiovascular, respiratory, and endocrine systems. These various changes ensure that the maternal body maintains a normal homeostasis that is suitable for both the mother and offspring. Pregnancy is also characterized by a progressive increase in nutrient-stimulated insulin responses, despite only a minor deterioration in glucose tolerance, consistent with progressive insulin resistance.

Changes in glucose metabolism occur to ensure the fetus’s growth and development, but also to balance maternal nutrition through all the pregnancy trimesters. During early pregnancy, glucose tolerance is normal or slightly improved as compared with postpartum [1]. The hyperinsulinemic-euglycemic glucose clamp technique indicates an increased sensitivity to the lowering of the blood glucose effect of exogenously administered insulin in the first trimester, compared to the second and third trimesters [2]. 

Insulin responses to an oral glucose load are also greater in the first trimester than before pregnancy, but in late pregnancy, glucose-tolerance tests have indicated that insulin action in late normal pregnancy is 50–70% lower than in nonpregnant women [3]. A progressive increase in basal and postprandial insulin concentrations is observed with advancing pregnancy. By the third trimester, basal and 24 h mean insulin concentrations may double [3]. The first and second phases of insulin release are 3- to 3.5-fold greater in late pregnancy [4].

The increase in maternal blood volume at the beginning of pregnancy causes a dilutional effect and, therefore, a partial drop in fasting blood glucose can be observed. Glucose levels remain constant in the second trimester; however, this is followed by a subsequent drop at the end of the pregnancy among healthy pregnant patients [5,6]. The glucose from maternal circulation is transferred through the fetal–placental unit, which, therefore, leads to a decline in its levels. To regulate this phenomenon, maternal insulin sensitivity decreases and hepatic gluconeogenesis and fatty acid levels increase [5].

In the first week of pregnancy, the fetal–placental unit causes a decrease in growth hormone levels, which promotes elevated insulin sensitivity. However, in the subsequent weeks of pregnancy, human placental lactogen (HCS), progesterone, cortisol, prolactin, and placental-derived human growth hormone (GH-V) compensates, which results in the observed decrease in insulin sensitivity during the second and third trimesters of pregnancy [7]. These hormonal changes observed in late pregnancy, such as rising concentrations of HCS, prolactin, cortisol, and glucagon, exert anti-insulinogenic and lipolytic effects that promote the greater use of alternative fuels, especially fatty acids, by peripheral tissues [8,9].

Studies on the role of cytokines produced by the adipose tissue and the production of inflammatory mediators by the placenta (e.g., tumor necrosis factor-α) have also shown their impact on tissue insulin sensitivity [5,10,11]. The elevation of post-prandial glucose levels may result from impaired insulin activity, as well as pancreatic β-cell-mediated insulin secretion and hepatic gluconeogenesis [5,12]. Prolactin and HCS increase the size and number of pancreatic beta cells. As the pregnancy continues, changes in the glucose metabolism and insulin sensitivity require the pancreas to produce more insulin. Inadequate production results in poor glycemic control [13,14,15].

Hyperglycemia in pregnancy (HIP) is one of the most common challenges in prenatal care. When first detected during pregnancy, it is classified either as diabetes mellitus in pregnancy (DIP) or gestational diabetes mellitus (GDM), depending on the time of diagnosis. It is believed that HIP occurs due to pre-existing diabetes, not detected before pregnancy, or because of insulin resistance that developed during pregnancy [16,17,18]. The Hyperglycemia and Adverse Pregnancy Outcome (HAPO) study in 2008 showed that hyperglycemia in pregnancy is less severe than overt diabetes, and it is independently associated with an increased risk of adverse maternal and neonatal outcomes [19].

HIP is a heterogeneous disorder, in which age, obesity, and genetic background contribute to the severity of the disease. HIP is accompanied by alterations in fasting, postprandial, and integrated 24 h plasma concentrations of amino acids, glucose, and lipids. These changes include a 3-fold increase in plasma triacylglycerol concentrations during the third trimester of pregnancy, elevation of plasma fatty acids, delayed postprandial clearance of fatty acids, and elevation of the branched-chain amino acids [20]. Some of those changes may disappear after pregnancy, but HIP may result in various long-term health challenges, for both the mother and the child—development of T2DM or inborn predisposition to obesity [21]. HIP may also result in abortion/miscarriage, development of hypertension, improper fetal growth (large for gestational age (LGA)/macrosomia), preterm birth, or intrauterine fetal death [22].

The prevalence of HIP and its complications is significantly associated with increased body-fat percentage, family history of T2DM, previous delivery of babies ≥ 4000 g, and the presence of symptoms of T2DM. Sedentary lifestyle, dietary factors, cigarette smoking, excessive pregnancy weight gain and high-body-fat accumulation also contribute to the risk of HIP [23,24,25,26].

Women with a history of DIP and GDM are more likely to have cardiovascular risk factors, including metabolic syndrome, T2DM, the occurrence of cardiovascular disease, as well as its complications, such as hypertension and ischemic heart disease at a younger age [27,28]. The American Heart Association includes a history of GDM in their classification of cardiovascular risk factors in women [29].

Intrauterine exposure to DIP and GDM is associated with a higher risk of abnormal glucose metabolism and adiposity in the offspring. Whether this is the impact of glycemia or maternal obesity is controversial. Kim et al., as well as Philips et al., proved that maternal hyperglycemia, independently of maternal BMI, is a risk factor for obesity or overweight in offspring [30,31]. Lowe et al. demonstrated that the offspring of mothers with untreated GDM are at high risk for impaired glucose tolerance 10–14 years postpartum [32]. There are studies connecting DIP and GDM with neurocognitive development, mental/psychomotor development, and overall intellectual function in offspring [33].

Glucose is the primary substrate for fetal oxidative metabolism, making its efficient transfer across the placenta essential for normal fetal growth and development. Insulin resistance in normal pregnancy increases glucose availability for the fetus. Insulin resistance is exacerbated in pregnancies complicated by any kind of diabetes, and maternal hyperglycemia leads to fetal hyperglycemia. There is an increased production of fetal insulin, insulin-like growth factor 1, and leptin, resulting in the stimulation of fetoplacental growth for mothers with gestational diabetes.

Cellular glucose uptake is a fundamental process for metabolism, growth, and homeostasis. Concentrative and facilitative glucose transporters are responsible for the movement of glucose across the plasma membrane. Glucose transport into cells depends on carrier proteins localized in the plasma membrane.

The placenta itself cannot produce appreciable amounts of glucose until late in gestation. Therefore, the uptake of maternal glucose is essential for glycogen synthesis. Members of the GLUT family facilitate glucose transfer [34]. In humans, there are three families of secondary glucose transporters: sodium-independent glucose transporters (GLUT proteins), encoded by SLC2A genes, sodium-dependent glucose transporters (SGLT proteins), encoded by SLC5A genes, and SWEET, encoded by the SLC50A gene [35]. Changes in the expression of the genes and/or the function of these transporters may be a factor in the development of various diseases and complications. 

It is important to consider the localization, function, and evolution of placental glucose transport systems, as they are altered with fetal development and the transport and the metabolic changes observed in pregnancy are complicated with maternal hyperglycemia.

## 2. Materials and Methods

To prepare this non-systematic narrative review, PubMed database was searched for relevant references from the first records until February 2022, using the following terms: (“placenta” AND “glucose transport”), (“placenta” AND “glucose transporter”), (“placenta” AND “GLUT”). The terms mentioned above were also searched with the phrase “trophoblast” instead of the “placenta”. At first, we excluded articles not related to the aims of the review, analyzing the titles and abstracts. Then, we checked the full texts of each paper included in this review. We applied the species searching filter—“Humans”. We selected only articles written in English. The first author (R.S.) was responsible for the searching and checking process.

## 3. Glucose Transport in the Human Placenta

### 3.1. Glucose Transporters Family

The human placenta is a complex, multi-functional structure, responsible for the exchange of gases and metabolic waste products and the supply of glucose to fetal circulation. The placenta is comprised of maternal (decidua basalis) and fetal origin tissues (trophoblastic and non-trophoblastic cell populations). The specialized transport membrane system separates the maternal circulation from the fetal one. The syncytiotrophoblast layer covers the chorionic villi and separates the intervillous pool of maternal blood from the fetal circulation. The villous barrier consists of a microvillous plasma membrane (MVM), covering the villi from the maternal side, and the fetal-facing basal plasma membrane (BM). Glucose transport across the human placenta is regulated by the family of glucose transporter proteins (GLUTs), located at both sides of the placental barrier, which modulate the sodium-independent facilitated-diffusion of glucose molecules through the placental membranes with the concentration gradient [36,37,38,39]. The GLUTs family includes over a dozen different isoforms of glucose transporters. BM permeability and GLUT1 density on its surface are two of the most relevant regulators of glucose-facilitated diffusion [40,41]. In vitro studies have revealed that the transplacental glucose transport is asymmetrical [42,43]. At similar transmembrane glucose gradients, the glucose uptake and flux are significantly higher, with the gradient going from the maternal to the fetal side of the placental membranes. This fact could be elucidated by the differences in the transport capacity of microvillus and basal plasma membranes and the increased glucose utilization by the placental tissue [42,43]. There are no differences in flux for non-metabolizable glucose analogs [42].

#### 3.1.1. GLUT1

**Figure 1:**
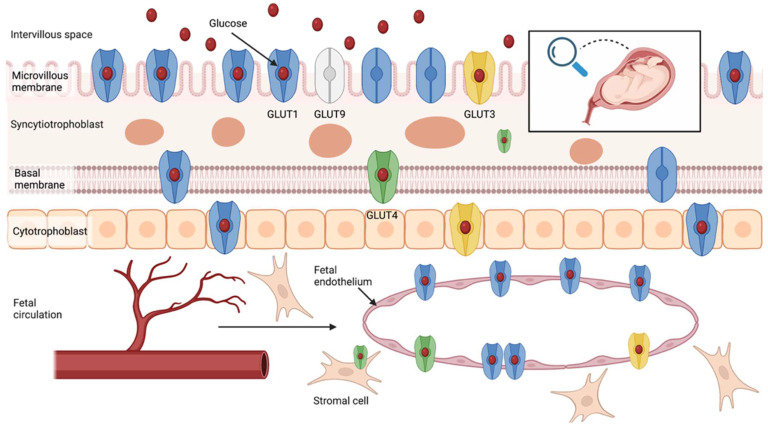
The distribution of glucose transporter (GLUTs) proteins across the human placenta. Own authorship—created with Biorender.com (accessed on 19 February 2022).

The GLUT1 protein is commonly expressed in numerous human tissues, including the placenta. The GLUT1 protein isoform plays a prominent role in regulating transplacental glucose transport. There have been attempts to establish its specific location across the placental tissue. Using immunohistochemistry, it was found that the GLUT1 expression is predominantly pronounced in the MVM of chorionic villi and less pronounced in the BM (Figure 1) [44]. It can also be detected in the plasma membrane of the villous and extravillous cytotrophoblast cells and placental endothelium from the early first trimester of pregnancy [37,43,44,45,46,47,48,49,50,51,52]. Studies utilizing in-situ hybridization techniques have confirmed the presence of GLUT1 mRNA in early pregnancy (16–22 weeks) and term placental explants. While Sakata et al. detected an increase in placental GLUT1 mRNA expression across pregnancy, other authors reported that GLUT1 mRNA expression is higher in early pregnancy explants, relative to term placentas [53,54]. GLUT1 mRNA was predominantly located in the syncytiotrophoblast cells. Its expression in non-syncytial cells was much less pronounced [55]. Further, the scientists using the Western blot technique confirmed the GLUT1 protein expression (~50–55 kDa) on both sides of the syncytiotrophoblast barrier (MVM and BM), cytotrophoblast cells, and in the fetal endothelium [37,48,56,57,58,59,60]. GLUT1 protein expression in the BM and MVM can be detected from early pregnancy. While MVM protein expression remains at relatively similar levels from 16 weeks to term pregnancy, BM GLUT1 protein expression may increase across the gestation [43]. According to current knowledge, the BM content in the GLUT1 transporters is a primary domain, limiting the transplacental glucose flow [61]. Michelsen et al. reported that neither MVM nor BM GLUT1 protein density are correlated with fetal or placental glucose consumption [58]. Interestingly, the placental GLUT1 mRNA expression was positively correlated with maternal age and inversely correlated with placental weight [62]. Finally, placental GLUT1 protein expression was positively correlated with the pre-pregnancy maternal BMI and umbilical artery glucose levels, but was not associated with the umbilical insulin levels [58,63].

#### 3.1.2. GLUT3

Human GLUT3 protein provides a high-affinity glucose transport to crucial organs and tissues highly dependent on constant glucose supply, even during hypoglycemia [64]. GLUT3 presence in the placental syncytial cells was confirmed at all gestational ages using immunohistochemistry [65]. GLUT3 is also expressed from the first trimester in MVM and villous cytotrophoblast cells [44,46,51,66,67,68,69]. Other reports revealed the presence of GLUT3 protein expression in the placental vascular endothelial cells [70,71,72]. In contrast, several authors did not detect the presence of GLUT3 in placental tissue sections using immunohistochemistry or immunofluorescence microscopy [43,73]. Multiple other studies confirmed the placental expression of GLUT3 mRNA [55,62,66,70]. GLUT3 mRNA expression could be detected in the early pregnancy placental tissue, and its expression remains unaltered in term explants [55]. In contrast to GLUT1, GLUT3 mRNA was detected at similar levels in syncytial, endovascular endothelium, and other types of placental cells [55,70]. Its cellular distribution is unaltered in early pregnancy and term placental samples [55]. GLUT3 protein (49 kDa) expression was detected in the placental membranes (MVM, BM) and intraplacental endothelial cells, but not in the isolated trophoblast cells [59,65,70,74]. Brown et al. reported that the MVM GLUT3 protein expression is the highest in the first trimester of pregnancy and decreases in the following trimesters, suggesting its essential role in the early trophoblast development [65]. Interestingly, several other authors did not observe the band representing the GLUT3 protein expression in the electrophoresis [43,56,60,73]. Hahn et al. detected the co-expression of glycogenin with GLUT3 in the placental tissue, and they speculated that GLUT3 might be responsible for regulating placental glycogen metabolism [72]. 

#### 3.1.3. GLUT4

The GLUT4 isoform provides the insulin-dependent glucose transport in various insulin-sensitive organs and tissues, such as the liver, adipose tissue, and muscles. The first findings suggested the lack of insulin-dependent GLUT4 glucose transporters expression in the placental tissue [71,73]. Subsequently, immunohistochemical analysis performed using the sections of healthy placenta revealed GLUT4 was mainly expressed in the fetal-facing BM of the syncytiotrophoblast rather than the MVM [37]. GLUT4 signal was also detected in the cytosol of term syncytial cells, and intravillous stromal and endothelial cells [46,75,76]. Interestingly, GLUT4 expression in the placenta is at levels averaging 25% of those detected in white adipose tissue [75]. GLUT4 mRNA expression was confirmed in the first trimester and term placental tissue [46,66,77]. It may be 10-fold lower than the adipose tissue GLUT4 mRNA expression [78]. While James-Allan et al. reported that the GLUT4 protein expression was 84% higher in the samples of BM collected from term placentas compared to early gestation samples collected from pregnancy terminations (11 to 24 weeks), Ericsson et al. detected much less pronounced GLUT4 (48 kDa) protein expression in term placental explants compared to the first trimester samples [37,46]. Xing et al. detected the colocalization of GLUT4 with the insulin receptors in the term human placental tissue.

#### 3.1.4. GLUT9 and Other Isoforms

Several other potentially less important GLUTs have also been identified in the fetomaternal tissues. For example, GLUT9 mRNA and protein expression (59 kDa) was detected in human placenta samples. Immunostaining analysis performed on the term placental explants revealed that GLUT9 is predominantly expressed by the syncytial cells. Its presence is especially pronounced in the microvillus membrane [57,79].

Both the immunoblotting and immunohistochemistry performed by Jansson et al. did not detect the expression of GLUT2 protein in placental tissue samples [55].

Gude et al. detected the GLUT12 mRNA in the term placental explants and confirmed GLUT12 protein expression through immunohistochemistry. GLUT12 protein expression was mainly pronounced in the first trimester syncytial and extravillous trophoblast cells and term villous vascular smooth muscle cells, villous stromal cells and trophoblast cells of the chorion [80,81]. Further, Stanirowski et al. confirmed GLUT8 and GLU12 protein expression in the term placental tissue by immunohistochemistry [82,83]. 

### 3.2. Potential Regulators of Glucose Transport in the Human Placenta

**Figure 2:**
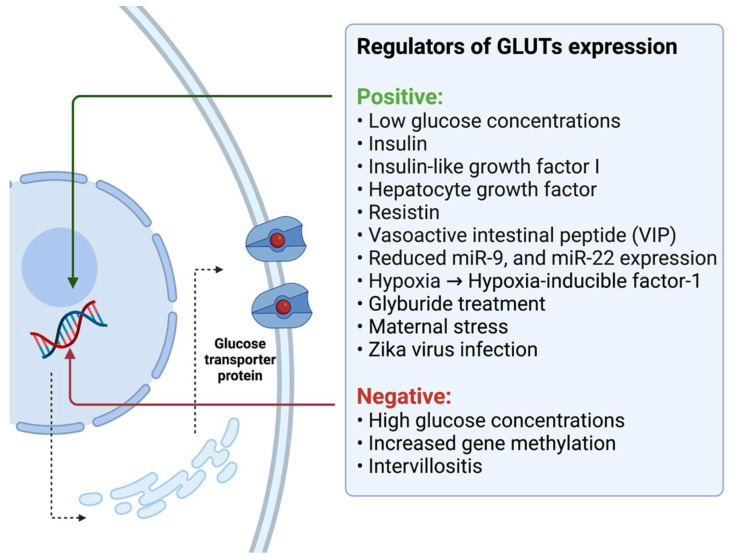
The potential regulators of GLUTs expression. Own authorship—created with Biorender.com (accessed on 19 February 2022).

Experimental studies discovered that several molecules could enhance or impair glucose transport mechanisms in the human placenta. The placental glucose uptake is increased in the presence of insulin and sodium nitroprusside, the nitric oxide (NO) donor. The nitric oxide effect is evoked by the stimulation of the guanylate cyclase signaling pathway—its blockade by the guanylate cyclase inhibitor completely inhibits the NO glucose uptake stimulation. However, it does not influence the insulin activity, suggesting the independent mechanisms of action in those molecules [84]. Insulin stimulation significantly increases the glucose uptake and GLUT1 expression in the first-trimester placental fragments but does not influence the glucose transport in the term explants (Figure 2) [46,85]. GLUT1 mRNA and protein expression and glucose uptake could also be altered by the various glucose concentrations [53,85,86,87,88]. Extremely high extracellular glucose concentrations hamper glucose uptake in syncytial cells and promote the internalization of plasma membrane GLUT1, where the temporary incubation in the absence of glucose stimulates GLUT1 expression [86,88,89]. Interestingly, James-Allan et al. discovered that in vitro stimulation of the placental tissue with insulin promotes the GLUT4 protein expression in BM (up to 77%) in early gestation and term placental explants. It also stimulates the phosphorylation of protein kinase B (Akt) at T303 and S473; however, it does not alter the overall Akt expression. They found no changes in GLUT1 expression after insulin stimulation [37].

DNA methylation patterns may also regulate the placental GLUTs’ expression across the pregnancy. While there was no evidence that DNA methylation could influence the expression of GLUT1 protein, Novakovic et al. noted that the epigenetic modifications modulate GLUT3 and GLUT10 expression. They discovered that the increasing gene methylation in the GLUT3 promoter region was associated with the decrease in its expression, observed along with the pregnancy progression [90]. Further, specific miRNAs, such as miR-9 and miR-22, were linked to the regulation of GLUT1 expression in patients with GDM. The reduction in their expression caused the increase in GLUT1 protein expression and could be responsible for the disturbances in glucose metabolism in patients with GDM [91].

Baumann et al. tried to evaluate the role of insulin-like growth factor-I (IGF-I) in the regulation of transepithelial glucose transport in the human placenta. Based on the outcomes of studies linking fetal growth with the IGF-I expression, they hypothesized that IGF-I could be responsible for the stimulation of GLUT1 expression and, as a consequence, promote the increased fetomaternal glucose transport that plays a role in the development of fetal macrosomia [61]. In the primary analyses, they used the population of BeWo choriocarcinoma cells as a model for trophoblast tissue. Their initial outcomes indicated that the treatment of BeWo and primary syncytial cells with increasing concentrations of IGF-I resulted in the increased expression of GLUT1 protein. It also promoted the transepithelial glucose transport across the BeWo monolayer (two-fold increase) and stimulated the glucose uptake into BeWo cells [61]. The experiment conducted on the placental tissue explants revealed that placental samples showed increased GLUT1 expression in BM specimens after the IGF-I treatment, but the MVM GLUT1 protein expression remained unchanged. The functional analysis concluded that the IGF-I perfusion of placental explants significantly elevated the GLUT1 protein expression in the BM. In contrast, the GLUT1 expression in the MVM was reduced [61]. Moreover, the BM GLUT1 and glucose transporter activity were correlated with fetal circulating IGF-I in healthy participants, while there was no association for patients with diabetes [92]. Further, the treatment of BeWo cells with 8-bromo-cyclicAMP promoted the increased glucose uptake, as well as the GLUT1 protein and mRNA expression [67]. In vitro experiments discovered that the GLUT1 expression and glucose uptake in placental explants can also be increased by the hepatocyte growth factor (HGF) [93]. 

The development of several pregnancy pathologies, such as fetal growth restriction and preeclampsia, is linked with predominant cellular hypoxia. Interestingly, hypoxia was proposed as the next condition associated with alterations in GLUTs protein expression. The induced hypoxic conditions significantly stimulated the GLUT1 and GLUT3 expression in BeWo choriocarcinoma cells in vitro and promoted transepithelial glucose transport. Hypoxia exerts its effects by increasing the synthesis of hypoxia-inducible factor-1 (HIF-1), which directly stimulates the increase in the GLUTs protein expression [94,95,96,97,98]. Nevertheless, Baumann et al. observed only a minimal increase in GLUTs expression under hypoxia in placental villous explants [94]. Interestingly, there was no changes in GLUTs (1, 4) expression in the placentas of women living at high altitude (>2500 m) compared to residents at low altitude (<1700 m) [99]. In contrast, Zamudio et al. detected significantly lower GLUT1 expression in the placental BM of women who live at high altitudes compared to those who live at normal altitudes [100,101]. They hypothesized that the lower fetomaternal glucose transport, caused by the preferential anaerobic glucose metabolism rather than chronic hypoxia, might be responsible for the lower birth weight of babies born at high altitudes. Finally, Francois et al. noted increased GLUT1 protein expression in women exposed to chronic placental hypoxia due to living at high altitudes [102]. Not only hypoxia but also placental intervillositis may alter the GLUTs expression. While inflammation caused by the plasmodium infestation decreases the GLUT1 expression, Zika virus infection was linked with the increase in the glucose uptake and GLUT3 expression in the first-trimester cytotrophoblast cells [103,104]. It was proposed that even maternal stress could alter the GLUTs expression [105].

The treatment of cytotrophoblast cells with the resistin could promote the increased placental glucose uptake by stimulating the GLUT1 mRNA and protein expression. Moreover, observed effects in response to glucose treatment could be associated with the increased ERK 1/2 phosphorylation in the analyzed cells after the resistin stimulation [106]. Further, the vasoactive intestinal peptide (VIP) has the capacity to promote the GLUT1 expression and glucose transport in human cytotrophoblast cells through the mammalian target of the rapamycin (mTOR) signaling pathway [107]. Furthermore, the inhibition of mTOR signaling resulted in a significant reduction in GLUT3 mRNA and protein expression in the JEG-3 human choriocarcinoma cell line [108]. It was speculated that the glucocorticoids might alter the placental GLUTs expression. While Hahn et al. reported that triamcinolone treatment decreased the GLUT1 and GLUT3 protein expression in the cultured term placental trophoblast cells, other authors found that it may promote GLUT1 and GLUT3 mRNA and protein synthesis in the in vitro cultured placental endothelial cells [109,110,111]. Another study discovered that treating cultured placental trophoblast cells with the corticotropin-releasing hormone (CRH) elevates GLUT1 and hampers GLUT3 expression [112]. In contrast, Ericsson et al. reported that the glucose transporter activity in the first trimester and term placentas are not affected by the short exposures to the numerous hormones and adipokines, such as insulin, leptin, cortisol, growth hormone, prolactin, and IGF-I [113].

Finally, several synthetic substances associated with glucose metabolism are suspected as potentially hazardous for proper early pregnancy development. For example, in vitro exposure to bisphenol A alters the GLUTs protein expression in human trophoblast cell models [114,115]. Whereas it increased the GLUT1 protein expression in the placental tissue collected from women with normal BMI, GLUT1 density in overweight patients was significantly decreased [77]. Furthermore, the expression of GLUT1 protein is significantly altered in the term syncytiotrophoblast membranes (MVM and BM) in GDM patients treated with glyburide compared to those collected from diet-treated patients. The increased GLUT1 expression was also detected in the population of in vitro cultured primary trophoblast cells treated with glyburide. Thus, the glyburide treatment could promote increased transplacental glucose transport and eventually lead to fetal overgrowth [116]. Glucose uptake and GLUT1 expression can also be downregulated by the oxidative stress evoked by the hypoxanthine–xanthine oxidase through the SIRT1-dependent mechanism [117].

## 4. Placental Glucose Transport in Patients with Diabetes

### 4.1. Pregestational Diabetes Mellitus (PGDM)

Patients with PGDM have significantly higher differences between the maternal blood glucose levels and fetal venous blood obtained from the umbilical vein immediately after delivery, compared to women with GDM and normoglycemic patients. Those discrepancies can represent the differences in placental glucose utilization, which lead to fetal growth abnormalities [118]. Patients with PGDM have an approximately two-fold increase in BM GLUT1 protein expression compared to normoglycemic women, shown in Table 1 [56,92,119]. Interestingly, there were no differences in the MVM GLUT1 protein expression in patients with PGDM and control individuals [56,92,119]. Sciullo et al. found no differences in the GLUT1 and GLUT3 mRNA placental expression between the populations of patients with diabetes and healthy controls [62]. Both PGDM and GDM patients treated with insulin had significantly increased GLUT4 and GLUT9 protein expression compared to healthy controls [120]. Finally, there were no differences in the GLUT3, GLUT8, and GLUT12 protein expression in patients with PGDM and control pregnant women [82].

Furthermore, the increased BM GLUT1 and decreased GLUT4 protein expression have been noted in the term placental samples obtained in patients with type 2 diabetes (T2D) compared with the healthy controls. Interestingly, in the same study, the MVM GLUT1 protein expression was inversely correlated with the percentage of neonatal fat mass. Nevertheless, the GLUTs protein expression has not been correlated with the fetal birth weight [121].

Multiple experimental studies focused on analyzing differences in functional consequences of alterations in the GLUTs expression in patients with diabetes. For instance, the prepared syncytiotrophoblast membranes (BM and MVM) were used to measure the glucose transport activity on both sides of the fetomaternal barrier. While their findings indicate that the glucose uptake across the BM could even be 60% higher in the diabetic population compared to the healthy individuals, there were no differences for the MVM [56,119]. Jansson et al. detected those BM functional differences in the glucose uptake, using the radiolabeled tracers, in the population of women with only moderately elevated HbA1c levels in the first trimester of pregnancy. Thus, those differences in the GLUTs expression and increased glucose uptake may partially elucidate the pathogenesis of fetal overgrowth in patients with relatively well-controlled White class D diabetes, which is often associated with vascular abnormalities that could, rather, lead to fetal growth restriction [119]. Finally, the placental GLUTs (1, 4, and 9) protein expression was positively correlated with the fetal birth weight and neonatal subscapular and abdominal fat mass measurements [63,82]. 

### 4.2. Gestational Diabetes Mellitus (GDM)

Several studies analyzed glucose transporters’ mRNA and protein expression in the population of patients with GDM. Women with GDM, diagnosed according to the International Association of the Diabetes and Pregnancy Study Groups (IADPSG) criteria, had markedly increased GLUT1 mRNA and protein expression compared to patients with normal glucose tolerance [47,91,123,127,128]. In contrast, other authors found no differences in GLUT1 mRNA or protein expression in women with GDM and control pregnant individuals [122,124,126]. Furthermore, Jansson et al. did not detect differences in the glucose uptake in term syncytial tissue from GDM and normal control pregnancies [122]. Insulin treatment might increase fetomaternal glucose transport in patients with GDM [127,129]. However, several experimental studies did not find alterations in GLUTs expression in GDM patients treated with insulin. Colomiere et al. indicated that diet-controlled GDM does not influence the GLUTs expression. The alternations in the GLUTs expression are often detected in the early pregnancy placental samples, but the GDM typically develops later in gestation and does not affect the early placentation. Moreover, the diet-controlled GDM is characterized by only moderately elevated fasting and post-prandial glycemic levels. Multiple in vitro studies found that GLUTs expression is exclusively altered in extremely hypo- or hyperglycemic conditions [53,85,86,87,88]. Thus, the mild glycemic changes in diet-controlled GDM do not affect placental GLUTs expression. GLUTs protein and mRNA expression are not always highly correlated [123]. Another study reported no differences in the GLUT1 MVM protein expression between the groups of diet- and insulin-controlled women with GDM and healthy controls [56]. Dekker et al. discovered significantly increased GLUT3 and GLUT4 mRNA expression in GDM patients compared to the controls [124]. Rong et al. discovered a decreased GLUT3 gene methylation and increased mRNA expression in GDM patients compared with control pregnant women [125]. Other studies found no differences in the placental GLUT4 or GLUT9 protein and mRNA expression between GDM and control pregnant women [47,75,78,91]. Further, there were no between-group differences in the GLUT8 and GLUT12 protein expression in patients with GDM and control pregnant women [82]. Zhang et al. noted significantly decreased GLUT3 and GLUT4 expression in non-obese GDM pregnant patients compared with normal pregnant women [126]. Finally, the placental GLUT4 protein density was positively correlated with the fetal birth weight in patients with insulin-dependent GDM [63].

## 5. Correlation with Fetal Growth and Other Gestational Outcomes

### 5.1. Fetal Macrosomia and Large-for-Gestational-Age (LGA) Newborns

Yao et al. revealed that the group of mothers of macrocosmic infants, who were diagnosed with GDM, had significantly increased GLUT1 mRNA and protein expression in the placental tissue compared with women with GDM and appropriate-for-gestational-age (AGA) infants, as well as the patients with normal glucose tolerance. Yao et al. also noted a significant positive correlation between neonatal birth weight and GLUT1 protein expression in each study subpopulation, shown in Table 2 [128]. In contrast, other authors noted decreased expression of GLUT1 in the term MVM samples but an increased GLUT1 protein expression in the term BM specimens [37]. Moreover, a negative correlation between neonatal birth weight and MVM GLUT1 protein content was found [56]. Other studies did not find differences in the BM GLUT1 protein expression between mothers of macrosomic babies and those with AGA infants in the population with diabetes [56,122]. Furthermore, Jansson et al. reported that both GLUT1 placental protein expression and glucose uptake were unaltered in GDM patients with LGA babies compared to the normoglycemic population [122]. 

Gaither et al. emphasized that increased GLUT1 expression and glucose uptake across the BM in patients with diabetes persisted, even though there was no evidence for the inappropriate current or recent long-term glycemic control among those patients (HbA1c values within the normal range). They hypothesized that long-lasting diabetes alters the fetomaternal glucose transport capacities and, in consequence, promotes glucose transport to the fetus, which stimulates excessive fetal growth [56]. Desoye et al. hypothesized that the occurrence of fetal macrosomia in women with adequate recent glycemic control might be associated with the impaired physiological placental capacity to store the excessive glucose in the form of glycogen [130]. 

**Table 2:** The summary of differences in the placental GLUTs mRNA and protein expression in pregnant patients with macrosomic babies and healthy control neonates.

Clinical Characteristics, Number of Study Participants	Analyzed Parameter	Main Findings	First Author; Year; [Reference]
Growth abnormalities, 6 IUGR vs. 6 macrosomia vs. 4 AGA from IDDM mothers vs. 8 controls	GLUT3 protein GLUT4 protein	No differences No differences	Kainulainen; 1997; [131]
Growth abnormalities, 8 PGDM and GDM with macrosomia vs. 17 diabetes with AGA	BM GLUT1 protein	No difference	Gaither; 1999; [56]
Growth abnormalities, 6 GDM with LGA vs. 32 controls	MVM GLUT1 protein BM GLUT1 protein	No difference No difference	Jansson; 1999; [122]
Growth abnormalities, 13 GDM with macrosomia vs. 13 GDM with AGA and 13 controls	GLUT1 mRNA GLUT1 protein	Increased Increased	Yao; 2017; [128]
Growth abnormalities, 5 obesity with macrosomia vs. 12 obesity with AGA and 11 controls (normal BMI)	MVM GLUT1 protein BM GLUT1 protein BM GLUT4 protein	Decreased Increased Decreased	James-Allan; 2019; [37]
Growth abnormalities; 26 with macrosomia vs. 20 controls	GLUT1 protein GLUT3 protein GLUT8 protein GLUT12 protein	No difference No difference No difference No difference	Stanirowski; 2021; [83]

Abbreviations: AGA—appropriate-for-gestational-age, BM—basal plasma membrane, BMI—body mass index, GDM—gestational diabetes mellitus, GLUT—glucose transporter, IDDM—insulin-dependent diabetes mellitus, IUGR—intrauterine growth restriction, LGA—large-for-gestational-age, MVM—microvillous plasma membrane, PGDM—pregestational diabetes mellitus.

The IGF-I protein may be involved in regulating placental glucose transport and the expression of GLUTs. Borges et al. aimed to analyze the consequences of alterations in the IGF-I axis for fetal growth. They found that circulating IGF-I, BM GLUT1 protein expression, and glucose transporter activity were correlated with fetal birth weight in the control group, but not in patients with diabetes. That fact could be elucidated by the detected decreased insulin-like growth-factor-binding protein (IGFBP) binding capacity in patients with diabetes compared with the controls. Interestingly, both patients with diabetes and healthy controls had HbA1c levels within the normal range. It is possible that increased GLUTs expression stimulates fetal growth, but there is a threshold limiting further excessive growth in patients with diabetes who reached the plateau [92]. In contrast, Balachandiran et al. observed the positive correlation between the GLUT1 placental protein expression and maternal circulating IGF-I concentrations and the fetal birth weight in patients with GDM [47]. Increased IGF-I and decreased adiponectin levels in patients with GDM might have been responsible for enhancing GLUT1 expression via the stimulation of placental insulin/IGF-1 signaling, which might have affected fetal growth [47].

Interestingly, the GLUT4 protein expression was significantly lower (up to 42%) in the BM samples obtained from mothers of macrosomic babies compared to the controls [37]. In contrast, Kainulainen et al. detected no between-group differences in the placental GLUT3 and GLUT4 protein expression in pregnancies affected by IUGR, fetal macrosomia, insulin-dependent diabetes, and healthy controls [131]. Finally, Stanirowski et al. did not find differences in the GLUTs (1, 3, 8, 12) protein expression in mothers of macrosomic babies and normal controls at term [83].

### 5.2. Expression of Glucose Transporters in Fetal Growth Restriction (FGR) and Other Pregnancy-Related Pathologies

Disturbances in fetal growth could be related to the altered expression of glucose transporters. Jansson et al. suggested that the intrauterine growth restriction (IUGR) incidence could be associated with the decreased placental GLUTs density in placental membranes. Nonetheless, there were no differences in the GLUT1 protein expression in placental tissue explants (BM and MVM) in control term membranes and two IUGR groups (term and preterm IUGR placental explants). Moreover, they did not find any differences in the glucose uptake between the IUGR samples and control placental membranes [43,132]. In contrast, Janzen et al. detected significant differences in the GLUT1, GLUT3, and GLUT4 mRNA expression in the term placental explant samples collected from patients with IUGR compared to healthy controls. However, utilizing the immunoblotting, they detected a significant increase only in the placental GLUT3 expression among patients with IUGR, but not in the healthy controls. Increased GLUT3 expression was associated with elevated HIF-1 levels, which rose in response to the disturbances in placental perfusion [66]. They also detected a significantly increased GLUT8 mRNA and protein expression in the samples obtained from the maternal side of the IUGR-affected placentas. Hypoxic conditions caused a further increase in GLUT8 expression in vitro [133]. Further, a significant increase in the placental GLUT1 and GLUT3 protein density was detected in placentas from pregnancies with fetal growth restriction (FGR) [83]. FGR is often associated with functional changes in placental blood flow and the incidence of preeclampsia. The laboratory analysis of placental villous explants revealed a decreased MVM GLUT1 protein expression and glucose transport capacity in samples obtained from pregnancies affected by preeclampsia compared to normal pregnancies [134]. Moreover, increased GLUT3 expression was detected in the preeclamptic placental tissue [135]. Interestingly, preeclampsia did not alter the BM GLUT1 expression and its transport activity [134].

Chang et al. assessed the GLUT1, GLUT3, and HIF-1 mRNA expression in the placentas collected from monochorionic twin pregnancies with selective growth restriction. The group of monochorionic pregnancies was divided into two subgroups—the first group with normal Doppler results and the second group diagnosed with absent or reverse-end diastolic flow in the umbilical artery within one week of delivery. They discovered significantly increased GLUT3 and HIF-1 expression in the samples obtained from patients with abnormal Doppler results [136]. They concluded that hypoperfusion, a characteristic feature of disturbances in placental blood flow, stimulates fetal glucose transport.

GLUT1 transcript and protein were also detected in the placental tissue in patients with intrahepatic cholestasis of pregnancy, in both cytotrophoblast and syncytial cells. There were no differences in GLUT1 mRNA expression in placentas of patients with cholestasis compared to that of the healthy controls. However, markedly higher GLUT1 protein density was detected in the cholestasis group [137]. 

Maternal obesity is associated with an increased risk of delivering a macrosomic infants. Multiple studies tried to elucidate the molecular mechanisms leading to excessive body weight in the infants of obese mothers. Ermini et al. did not find any differences in the GLUT1 and GLUT4 mRNA and protein expression in the placental explants obtained from overweight pregnant patients and pregnant women with normal BMI [77]. Colomiere et al. reported that maternal obesity does not alter GLUT1 and GLUT4 protein expression [123]. Furthermore, there were no differences in the early pregnancy (6–24 weeks) GLUT1 and GLUT3 protein expression in obese women and pregnant patients with normal BMI [59]. Acosta et al. investigated the potential correlation of GLUT1 and GLUT9 placental expression with the macrosomia incidence. They found that BM GLUT1 density was significantly positively correlated with the birth weight in infants of obese non-diabetic mothers [57].

Based on the murine models, in which Assisted Reproductive Technology (ART) application resulted in alterations in GLUTs expression, often leading to placental overgrowth, Dong et al. analyzed the GLUTs expression in human placentas in ART pregnancies. They discovered the increased GLUT (1, 3, 8, and 11) mRNA and GLUT1 protein expression in ART placental explants with unchanged mTOR activity. Nonetheless, there were no differences in placental and fetal birth weight in their study cohort, composed of ART and normal pregnancies [138]. In contrast, Bloise et al. found no between-group differences in the GLUT1 expression in pregnancies conceived by intracytoplasmic sperm injection and normal control pregnancies [139]. The expression of GLUTs can also be affected by the various preparation and embryo transfer techniques applied in ART procedures (summarized in Table 3) [140].

## 6. Conclusions

In summary, several types of transporters provide constant glucose transport to the developing fetus through the placental barrier from the early stages of pregnancy. GLUT1 is a prominent protein isoform involved in regulating placental glucose-transporting capacity. The GLUT1 membrane protein density and permeability of the syncytial BM are the main factors limiting the glucose-facilitated diffusion in the fetomaternal compartment in physiological conditions. Besides, GLUT1, GLUT3 and GLUT4 isoforms are widely expressed across the human placenta. 

There are multiple contradictory reports describing the GLUTs expression in pregnancies with diabetes. However, most studies indicated that diabetes upregulates the BM GLUTs density and, as a result, promotes fetomaternal glucose transport. It is believed that both PGDM and GDM affect the GLUTs placental expression. Whereas the alterations in GLUTs expression in patients with PGDM are detected from the first trimester and then persist throughout the gestation, the changes caused by GDM occur later in pregnancy and are less pronounced. Interestingly, the upregulated placental GLUTs expression and glucose uptake are often detected in relatively well-controlled PGDM patients with no evidence of recent chronic episodes of hyperglycemia, even in the first trimester samples. That phenomenon could partially elucidate the elusive nature of fetal growth abnormalities in women with long-lasting diabetes and appropriate glycemic control. In general, the increased nutrient transport, pronounced by increased GLUTs expression, should be associated with the pathogenesis of excessive fetal growth. Surprisingly, most studies found no between-group differences in GLUTs placental expression in macrosomic and normal control pregnancies. Further, numerous biologically active molecules, such as hormones, adipokines, and cytokines, modulate the GLUTs mRNA and protein expression. Therefore, the disturbances in their release can, in consequence, be responsible for the stimulation of excessive fetal growth.

The fetomaternal GLUTs expression may also be influenced by several other conditions, such as chronic hypoxia in pregnancies with abnormal Doppler results, preeclampsia, intrahepatic cholestasis of pregnancy, and pregnancies conceived by ART.

To date, the placental GLUTs expression has been determined in the samples collected after a term or preterm delivery and in patients who underwent the early planned pregnancy termination. There are no studies with complete follow-up assessing the early pregnancy GLUTs expression in connection with the analysis of pregnancy outcomes. Using the technique proposed by Imudia et al., our research team is planning to measure the GLUTs expression in the non-invasively obtained first-trimester extravillous trophoblast cells and analyze its potential influence on the regulation of fetal growth [141].

## Figures and Tables

**Table 1:** The summary of differences in the placental GLUTs mRNA and protein expression in pregnant patients with diabetes and heathy control individuals.

Clinical Characteristics, Number of Study Participants	Analyzed Parameter	Main Findings	First Author; Year; [Reference]
PGDM + GDM, 10 patients with diabetes (5 IDDM, 2 NIDDM, 3 GDM) vs. 9 controls	GLUT1 mRNA GLUT3 mRNA	No difference No difference	Sciullo; 1997; [62]
Pregestational diabetes, 6 PGDM vs. 7 controls	MVM GLUT1 protein BM GLUT1 protein	No difference Increased	Gaither; 1999; [56]
Pregestational diabetes, 7 White’s class D vs. 21 controls	MVM GLUT1 protein BM GLUT1 protein	No difference Increased	Jansson; 1999; [119]
PGDM + GDM, 12 IDDM (6 PGDM, 6 GDMA2) vs. 25 controls	GLUT4 protein GLUT9 protein	Increased Increased	Stanirowski; 2017; [120]
Pregestational diabetes, 6 PGDM vs. 25 controls	GLUT1 protein	Increased	Stanirowski; 2017; [120]
Pregestational diabetes, 14 T1D, class B vs. 19 controls	MVM GLUT1 protein BM GLUT1 protein	No difference Increased	Borges; 2019; [92]
Pregestational diabetes, 20 PGDM vs. 23 controls and 60 GDM	GLUT1 protein GLUT3 protein GLUT8 protein GLUT12 protein	Increased No difference No difference No difference	Stanirowski; 2021; [82]
Pregestational diabetes, 9 T2D vs. 9 controls	MVM GLUT1 protein BM GLUT1 protein BM GLUT4 protein	No difference Increased Decreased	Castillo-Castrejon; 2021; [121]
Gestational diabetes, 7 GDMA2 vs. 12 controls	GLUT4 mRNA GLUT4 protein	No difference No difference	Xing; 1998; [75]
Gestational diabetes, 16 GDM vs. 7 controls	MVM GLUT1 protein BM GLUT1 protein	No difference Increased	Gaither; 1999; [56]
Gestational diabetes, 18 GDM vs. 32 controls	MVM GLUT1 protein BM GLUT1 protein	No difference No difference	Jansson; 1999; [122]
Gestational diabetes, 6 GDMA2 vs. 7 controls (non-obese)	GLUT1 mRNA GLUT1 protein GLUT4 mRNA GLUT4 protein	No difference Increased Decreased Decreased	Colomiere; 2009; [123]
Gestational diabetes, 6 GDMA2 vs. 7 controls (obese)	GLUT1 mRNA GLUT1 protein GLUT4 mRNA GLUT4 protein	No difference No difference Decreased No difference	Colomiere; 2009; [123]
Gestational diabetes, 20 GDM vs. 18 controls	GLUT4 mRNA	No difference	Kuzmicki; 2011; [78]
Gestational diabetes, 19 GDM vs. 19 controls	GLUT1 mRNA GLUT3 mRNA GLUT4 mRNA	No difference Increased Increased	Dekker; 2014; [124]
Gestational diabetes, 36 GDM vs. 40 controls	GLUT3 mRNA	Increased	Rong; 2015; [125]
Gestational diabetes, 10 GDM vs. 10 controls	GLUT1 mRNA GLUT1 protein GLUT3 mRNA GLUT3 protein GLUT4 mRNA GLUT4 protein	No difference No difference No difference Decreased Decreased Decreased	Zhang; 2016; [126]
Gestational diabetes, 6 GDMA2 vs. 6 GDMA1 vs. 6 controls	GLUT1 mRNA GLUT1 protein	Increased in GDMA2 Increased in GDMA2	Muralimanoharan; 2016; [127]
Gestational diabetes, 26 GDM vs. 13 controls	GLUT1 mRNA GLUT1 protein	Increased Increased	Yao; 2017; [128]
Gestational diabetes, 24 GDMA2 vs. 19 controls	MVM GLUT1 protein BM GLUT1 protein	No difference Increased	Borges; 2019; [92]
Gestational diabetes, 31 GDM vs. 20 controls	GLUT1 mRNA GLUT1 protein GLUT4 mRNA GLUT4 protein GLUT9 mRNA GLUT9 protein	No difference Increased No difference No difference No difference No difference	Song; 2021; [91]
Gestational diabetes, 20 GDM vs. 20 controls	GLUT1 mRNA GLUT1 protein GLUT4 protein	Increased Increased No difference	Balachandiran; 2021; [47]
Gestational diabetes, 60 GDM vs. 23 controls	GLUT1 protein GLUT3 protein GLUT8 protein GLUT12 protein	No difference No difference No difference No difference	Stanirowski; 2021; [82]

Abbreviations: BM—basal plasma membrane, GDM—gestational diabetes mellitus, GLUT—glucose transporter, IDDM—insulin-dependent diabetes mellitus, MVM—microvillous plasma membrane, PGDM—pregestational diabetes mellitus, T1D—type 1 diabetes, T2D—type 2 diabetes.

**Table 3:** The expression of GLUTs in several maternal pregnancy-related complications and its association with fetal growth.

Medical Condition	GLUTs Expression; Main Findings	Association with the Fetal Growth
Pregestational diabetes mellitus	Multiple conflicting results— most likely increased GLUTs expression	No evidence of association with macrosomia. Positive correlation between GLUT1, GLUT3, and GLUT4 expression and fetal birth weight.
Gestational diabetes mellitus	Multiple conflicting results— less pronounced increase in GLUTs expression, more likely in insulin-dependent patients	Not enough evidence supporting association with macrosomia. Potential positive correlation between GLUT1, and GLUT4 expression and fetal birth weight.
Abnormal Doppler examination results suggesting the placental insufficiency	Compensatory increased GLUT3 protein expression stimulated by hypoxia	Association with the fetal growth restriction
Preeclampsia	Decreased MVM GLUT1 and increased GLUT3 expression	-
Maternal obesity	No differences in GLUT1, GLUT4 and GLUT3 placental protein expression in obese and control individuals	Positive correlation between BM GLUT1 expression and fetal birth weight in obese mothers
Intrahepatic cholestasis of pregnancy	Increased GLUT1 protein expression	-
ART pregnancies	Markedly altered GLUTs mRNA expression	No association

Abbreviations: ART—assisted reproductive technology, BM—basal plasma membrane, GLUT—glucose transporter, MVM—microvillous plasma membrane.

## Data Availability

Not applicable.
